# Safety and efficacy of transcatheter aortic valve replacement in rheumatic aortic regurgitation: a prospective cohort study

**DOI:** 10.3389/fcvm.2026.1662827

**Published:** 2026-02-18

**Authors:** Yaojie Wang, Yu Mao, Yang Liu, Mengen Zhai, Ping Jin, Yazheng Zhang, Xinbo Liu, Haibo Yang, Hua Zhang, Youjin Li, Jian Yang

**Affiliations:** 1 Department of Cardiovascular Surgery, People’s Hospital of NingXia Hui Autonomous Region, NingXia, China; 2 Department of Cardiovascular Surgery, Xijing Hospital, Xi’an, Shaanxi, China

**Keywords:** aortic regurgitation, high surgical risk, J-valve, rheumatic heart disease, transcatheter aortic valve replacement

## Abstract

**Background:**

The prevalence of rheumatic aortic regurgitation (AR) is higher than that of aortic stenosis in developing countries, but the efficacy of transcatheter aortic valve replacement (TAVR) in high surgical risk patients with severe AR remains unknown. Our goal was to explore the differences in clinical outcomes of TAVR in patients with rheumatic and non-rheumatic AR.

**Methods:**

144 Rheumatic and 417 nonrheumatic patients with severe AR were prospectively enrolled from January 2018 to December 2021. All patients underwent transapical TAVR with J-Valve after evaluation by computed tomography angiography and transthoracic echocardiography before the procedure. The primary end point was 3-year all-cause mortality.

**Results:**

The average age was 71.2 [interquartile range (IQR): 66.0–76.0] years, and the Society of Thoracic Surgeons score was 4.8 (IQR: 3.4–6.1) %. The proportion of patients with rheumatic AR who developed ≥ mild PVL was lower than the proportion of patients with non-rheumatic AR (5.6% vs. 11.3%, *P* < 0.001). At a median follow-up of 39.7 (IQR: 36.4–41.8) months, no difference was observed in the 3-year all-cause mortality (*P* = 0.740) between the two groups. After multivariate adjustment, the Society of Thoracic Surgeons score, higher frailty and larger aortic angulation were associated with 3-year all-cause mortality.

**Conclusion:**

For patients with rheumatic AR, the clinical outcomes were similar to those of patients with non-rheumatic AR. TAVR can be one of the feasible treatment options for such patients.

## 1 Introduction

Although rheumatic heart disease (RHD) is less prevalent in developed countries, it remains a major public cardiac burden in developing countries (1, 2). Although the mitral valve is most affected by RHD, 24.3%–31.5% of patients with RHD undergoing mitral valve surgery require aortic valve (AV) interventions during the same period (3–5).

As recommended in the guidelines, surgical aortic valve replacement (SAVR) is still the preferred treatment for rheumatic AV disease (6, 7). However, many patients with aortic regurgitation (AR) are still inoperable due to advanced age or comorbidities. Transcatheter aortic valve replacement (TAVR) has developed rapidly in recent years, which could achieve outcomes in patients with AR similar to those achieved in patients with aortic stenosis (AS) (8).

However, AV leaflets are usually accompanied by severe fibrosis in patients with rheumatic diseases, and calcification accumulates only in the late stages of the degenerative changes (9). Furthermore, anatomical and functional differences compared with degenerative AR may increase technical challenges on transcatheter heart valve THV release and implantation (10). Our goal was to provide evidence of the safety and efficacy of TAVR in patients with rheumatic AR. This study is the largest to date to explore differences of clinical outcomes in patients with rheumatic vs. non-rheumatic AR, so as to evaluate the safety and efficacy of TAVR in such patients.

## 2 Methods

### 2.1 Study design and population

**Figure 1:**
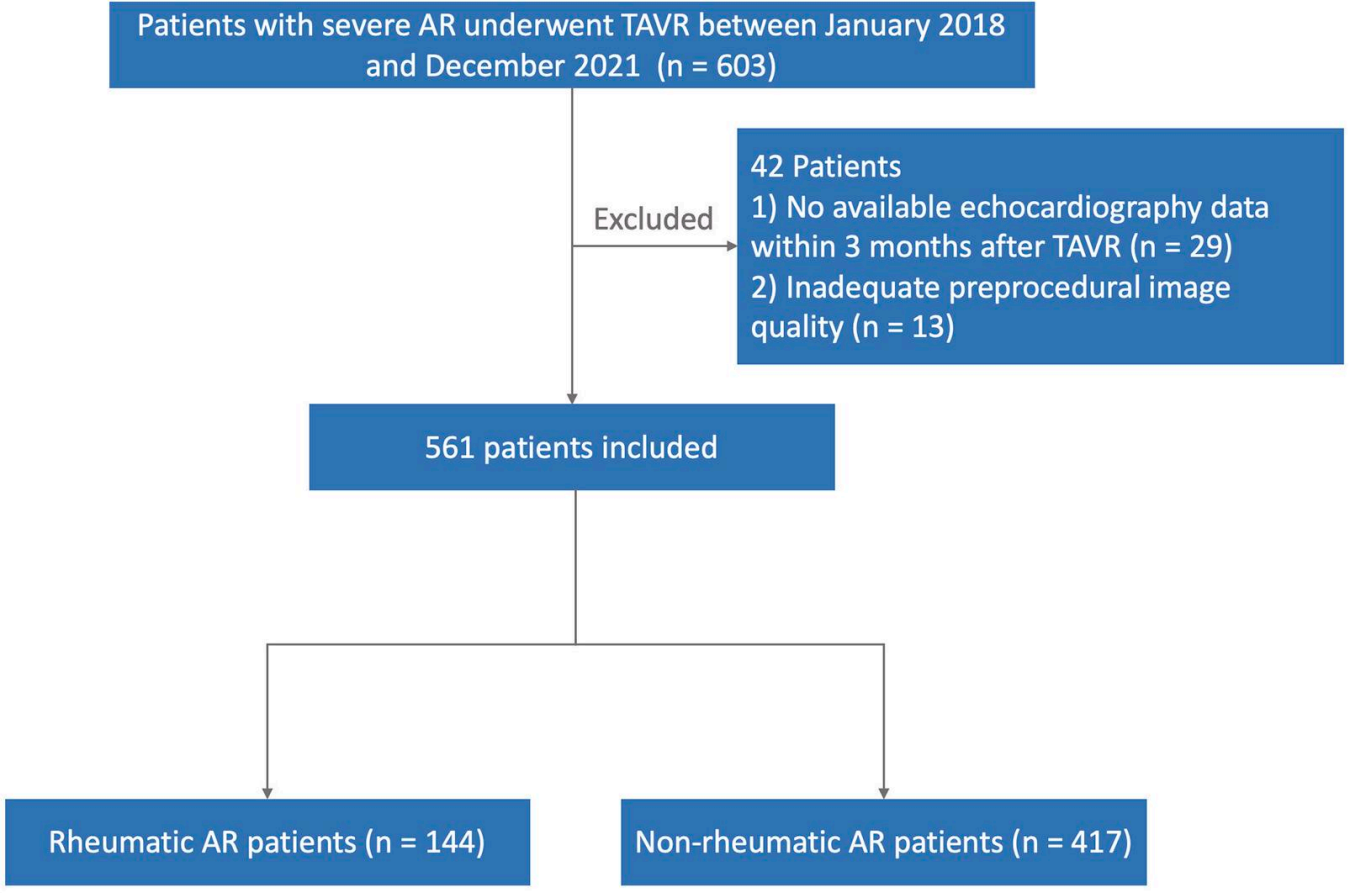
Flow chart. A total of 561 patients were divided on the basis of rheumatism.

This study prospectively included patients with severe AR in two high-volume centers from January 2018 to December 2021. The inclusion criteria included: (i) age ≥ 60 years; (ii) New York Heart Association functional class ≥ II; (iii) ≥ moderate AR diagnosed by transthoracic echocardiography; and (iv) European System for Cardiac Operative Risk Evaluation score II > 12% or Society of Thoracic Surgeons (STS) score > 8%. The exclusion criteria were: (i) < moderate AR; (ii) myocardial infarction within the past month; (iii) history of endocarditis; (iv) hypertrophic cardiomyopathy; and (v) transient ischemic attack/stroke within the past six months. We identified patients diagnosed with rheumatic AR using the International Classification of Diseases–10th Edition (ICD-10) (11). Patients were evaluated by a team of cardiac surgeons, echocardiographers and engineers. 105 clinical variables correlated with frailty were used to calculate frailty scores for all patients (12). 42 patients were excluded from the study: 29 of them had no available echocardiography data after TAVR, and 13 of them had inadequate quality of preprocedural imaging. Then, the study cohort was divided into two groups: patients with rheumatic AR (*n* = 144) and patients with non-rheumatic AR (*n* = 417) (Figure 1). This study complied with the Declaration of Helsinki and was approved by the local ethics commissions. All patients provided written informed consent for the procedures and for subsequent data collection.

### 2.2 Preprocedural assessment

2-Dimensional Imaging Assessment. Computed tomography angiography (CTA) and transthoracic echocardiography (TTE) were measured and evaluated before the procedure was carried out. The standard Digital Imaging and Communications in Medicine format for CTA data was imported into 3Mensio software (Materialise, Belgium) to assess the aortic root, including the annulus and the left ventricular outflow tract, with particular attention to the size, location, and position of the sinuses of Valsalva and the angle of the aorta. The TTE assessed atrioventricular morphology and the degree of AR, pressure gradient, left ventricular ejection fraction, left ventricular fractional shortening, and other preexisting cardiac complications.

### 2.3 Procedure and end points

The patient was given general anesthesia and intubated, and a catheter was inserted into the right internal jugular vein and a sheath for atrial pacing was implanted. After routine heparinization, a 4–6 cm surgical incision was made between the fifth and sixth intercostal spaces on the left midclavicular line. After the apex of the heart was fully exposed, the pericardium was incised and suspended. A sheath catheter was inserted into the femoral artery, and a braided catheter was placed at the bottom of the right/non-coronary sinus for intraoperative positioning. The delivery system was inserted into the atrioventricular along a rigid wire, and the *J*-Valve delivery system was inserted through the transapical approach. Then, the three positioning keys was opened to enter the sinus, and the THV was released with the support of the wire. The position and function of the THV were immediately evaluated using digital subtracted angiography and transesophageal echocardiography. The detailed procedures were described earlier (13).

Clinical outcomes were defined according to the Valvular Academic Research Consortium-3 criteria (14). The primary end point was the 3-year all-cause mortality. Secondary end points included a combination of all-cause mortality and rehospitalization for heart failure at 3 years. Additionally, TAVR-related complications and clinical outcomes in patients with co-existing AS were also examined.

### 2.4 Statistical analyses

All data were tested for normality and homogeneity of variance. Continuous variables were reported as mean ± standard deviation and compared using analysis of variance or the median [interquartile range (IQR)]. If the data were not normally distributed, the Mann–Whitney *U*-test was used. Categorical variables were reported as frequencies and compared using the Chi-square or the Fisher exact test, if applicable. Comparisons of continuous variables between the two groups included the analysis of variance or the Kruskal–Wallis test (as appropriate). The multivariate model included statistically significant variables with univariate analysis (*P* < 0.05). Logistic regression analysis results were expressed as the odds ratio (OR) and the 95% confidence interval (CI). Cumulative mortality was calculated using the Kaplan–Meier survival analysis. Bilateral *P*-values < 0.05 were considered statistically significant. Statistical analyses were performed using R programming language Version 4.2.2 (R Foundation for Statistical Computing, Vienna, Austria).

## 3 Results

### 3.1 Baseline characteristics

A total of 561 patients with AR who underwent TAVR were included: 417 patients with non-rheumatic AR and 144 patients with rheumatic AR. Table 1 shows the baseline characteristics of the two groups. Furthermore, there were 73 patients with rheumatic AR and 188 patients with non-rheumatic AR combined with AS (Supplementary Table S1). The average age was 71.2 (IQR: 66.0–76.0) years, and the Society of Thoracic Surgeons score for patients with rheumatic AR and non-rheumatic AR were 7.5 (IQR: 6.2–9.8) % and 6.6 (IQR: 4.3–8.8) %, respectively. Notably, although patients with rheumatic AR had lower frailty scores compared to patients with non-rheumatic AR [7.0 (IQR: 5.6–14.8) % vs. 10.6 (IQR: 8.3–14.3) %, *P* < 0.001], the proportion of patients with a high frailty score was higher (24.3% vs. 15.1%, *P* = 0.068).

**Table 1:** Baseline demographic characteristics.

Characteristics	Non-rheumatic AR (*n* = 417)	Rheumatic AR (*n* = 144)	*P-* Value
Age, years	71.0 (67.0–75.0)	72.0 (66.0–78.0)	0.676
Male	74.6 (311)	75.7 (109)	0.916
BMI, kg/m^2^	24.1 (21.2–26.3)	24.4 (22.1–27.3)	0.923
BSA, m^2^	1.7 (1.6–1.7)	1.7 (1.6–1.8)	0.704
NYHA functional class ≥ III	97.6 (407)	93.8 (135)	0.269
STS score, %	6.6 (4.3–8.8)	7.5 (6.2–9.8)	0.340
EuroSCORE, %	6.2 (4.0–9.0)	6.2 (3.8–9.3)	0.885
Diabetes mellitus	18.0 (75)	18.8 (27)	0.826
Hypertension	64.0 (267)	68.8 (99)	0.647
Dyslipidemia	16.3 (68)	17.4 (25)	0.837
Cerebrovascular disease	2.6 (11)	4.2 (6)	0.126
Coronary artery disease	27.6 (115)	26.4 (38)	0.724
PCI	2.9 (12)	4.2 (6)	0.167
CABG	1.2 (5)	2.8 (4)	0.140
Atrial ﬁbrillation	22.8 (95)	22.2 (32)	0.950
Previous PM	2.9 (12)	3.5 (5)	0.601
Peripheral artery disease	38.1 (159)	27.1 (39)	0.088
NT-proBNP	2,392.0 (1,736.0–3,020.0)	2,131.0 (1,414.0–3,093.5)	0.852
Frailty score	10.6 (8.3–14.3)	7.0 (5.6–14.8)	**<0**.**001**
High frailty	15.1 (63)	24.3 (35)	0.068

Values are expressed in interquartile range, or % (n). The *P*-values in bold represent differences between groups with *P*-values < 0.05.

AR, aortic regurgitation; TAVR, transcatheter aortic valve replacement; BMI, body mass index; BSA, body surface area; PCI, percutaneous coronary intervention; CABG, coronary artery bypass graft; PM, pacemaker; NYHA, New York Heart Association; STS, Society of Thoracic Surgeons; EuroSCORE, European system for cardiac operative risk evaluation; NT-proBNP, N-terminal pro–B-type natriuretic peptide.

### 3.2 Preprocedural TTE and CTA characteristics

Preprocedural imaging assessments are shown in Table 2 and Supplementary Table S2. There was no significant difference in TTE measurements among the groups, except for the higher proportion of patients with rheumatic AR having a bicuspid aortic valve (11.8% vs. 5.8%, *P* < 0.001). Although there was no significant statistical difference between the two groups, patients with rheumatic AR had a smaller annular area, annular diameter, left ventricular outflow tract diameter, sinotubular junction diameter, and ascending aorta diameter. Notably, the effective orifice area (EOA) and the indexed EOA were similar.

**Table 2:** Baseline echocardiographic and computed tomography characteristics.

Characteristics	Non-rheumatic AR (*n* = 417)	Rheumatic AR (*n* = 144)	*P-* Value
Transthoracic echocardiography			
BAV	5.8 (24)	11.8 (17)	**<0**.**001**
Mean pressure gradient, mmHg	6.9 (5.0–9.3)	11.6 (9.2–14.2)	0.936
LVEF, %	51.0 (44.0–56.0)	47.5 (43.0–56.5)	0.428
Combined with AS	45.1 (168)	50.7 (73)	0.144
≥Moderate MR	21.8 (91)	25.7 (37)	0.604
EOA, cm^2^	1.0 (0.9–1.3)	1.0 (0.8–1.2)	0.068
EOAi, cm^2^/m^2^	0.9 (0.7–1.1)	0.9 (0.7–1.0)	0.120
Computed tomography angiography			
Aortic annulus area, mm^2^	587.5 (518.8–629.7)	548.0 (506.5–611.0)	0.116
Aortic annulus diameter, mm	27.8 (26.3–28.7)	26.5 (25.2–27.4)	0.291
LVOT diameter, mm	29.3 (27.5–30.4)	28.4 (26.1–29.4)	0.335
STJ diameter, mm	34.3 (31.2–37.7)	33.5 (30.4–35.9)	0.372
AA diameter, mm	38.8 (36.1–40.7)	37.5 (35.0–40.2)	0.085
Aortic angulation, degree	44.0 (41.0–50.0)	45.0 (42.0–50.0)	0.421

Values are expressed in interquartile range, or % (n). The *P*-values in bold represent differences between groups with *P*-values < 0.05.

AA, ascending aorta; AR, aortic regurgitation; BAV, bicuspid aortic valve; EOA, effective orifice area; EOAi, indexed EOA; LVEF, left ventricle ejection fraction; LVOT, left ventricular outflow tract; MR, mitral regurgitation; STJ, sinotubular junction.

### 3.3 Procedural details and clinical outcomes

The data related to procedures are shown in Table 3 and Supplementary Table S3. All patients were treated by the transapical approach. The proportion of patients with oversized implant was higher in the rheumatic AR group than in the non-rheumatic AR group (18.8% vs. 7.2%, *P* < 0.001). Notably, the proportion of patients with rheumatic AR who developed ≥ mild PVL was lower than the proportion of patients with non-rheumatic AR (5.6% vs. 11.3%, *P* < 0.001).

**Table 3:** Procedural details and hospitalization characteristics.

Characteristics	Non-rheumatic AR (*n* = 417)	Rheumatic AR (*n* = 144)	*P-* Value
Bioprosthetic valve size			
<25 mm	11.5 (48)	25.0 (36)	**<0**.**001**
≥26 mm	88.5 (369)	75.0 (108)	**<0**.**001**
Implanted valve oversize	7.2 (30)	18.8 (27)	**<0**.**001**
Intraprocedural characteristics			
Conversion to open heart surgery	1.4 (6)	2.1 (3)	N/A
Annulus rupture	1.4 (6)	2.1 (3)	N/A
Coronary obstruction	0.2 (1)	1.4 (2)	N/A
Valve-in-valve implant	0.5 (2)	2.8 (4)	0.264
≥Mild paravalvular leakage	11.3 (47)	5.6 (8)	**<0**.**001**
Operation time, min	120.0 (105.0–125.0)	111.0 (85.5–139.5)	0.548
Fluoroscopy time, min	7.9 (5.9–10.3)	7.4 (5.9–10.4)	0.330
Periprocedural outcomes			
Stroke	0.7 (3)	1.4 (2)	0.302
Life-threatening bleeding	1.9 (8)	2.8 (4)	0.457
Major vascular complications	4.1 (17)	5.5 (8)	0.420
Acute kidney injury Staging ≥ 3	4.6 (19)	6.9 (10)	0.231
New-onset permanent pacemaker implantation	6.0 (25)	5.5 (8)	0.820

Values are expressed in interquartile range or % (*n*). The *P*-values in bold represent differences between groups with *P*-values < 0.05.

ICU, intensive care unit. N/A, not applicable.

**Figure 2:**
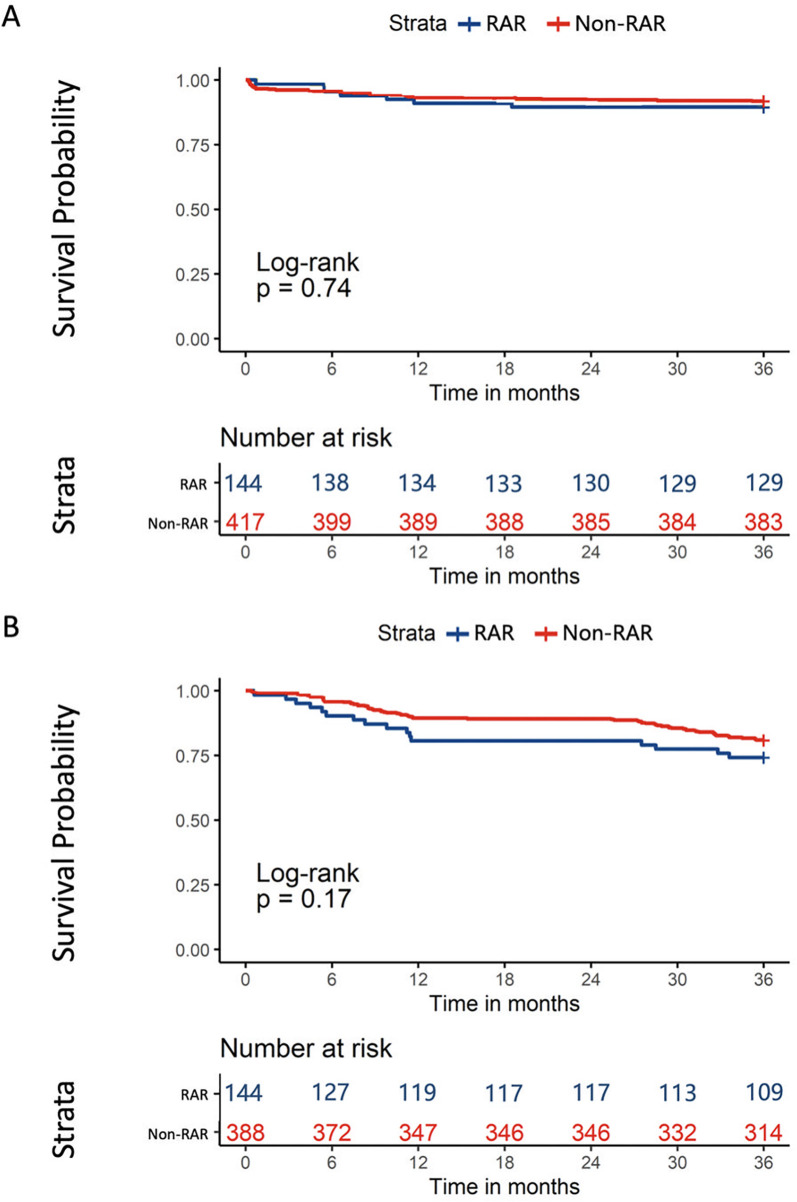
Kaplan–meier analysis of all-cause mortality **(A)** and composite end point **(B)** at 3 years comparing patients with rheumatic AR and non-rheumatic AR. AS, aortic stenosis; RAR, rheumatic aortic regurgitation.

**Figure 3:**
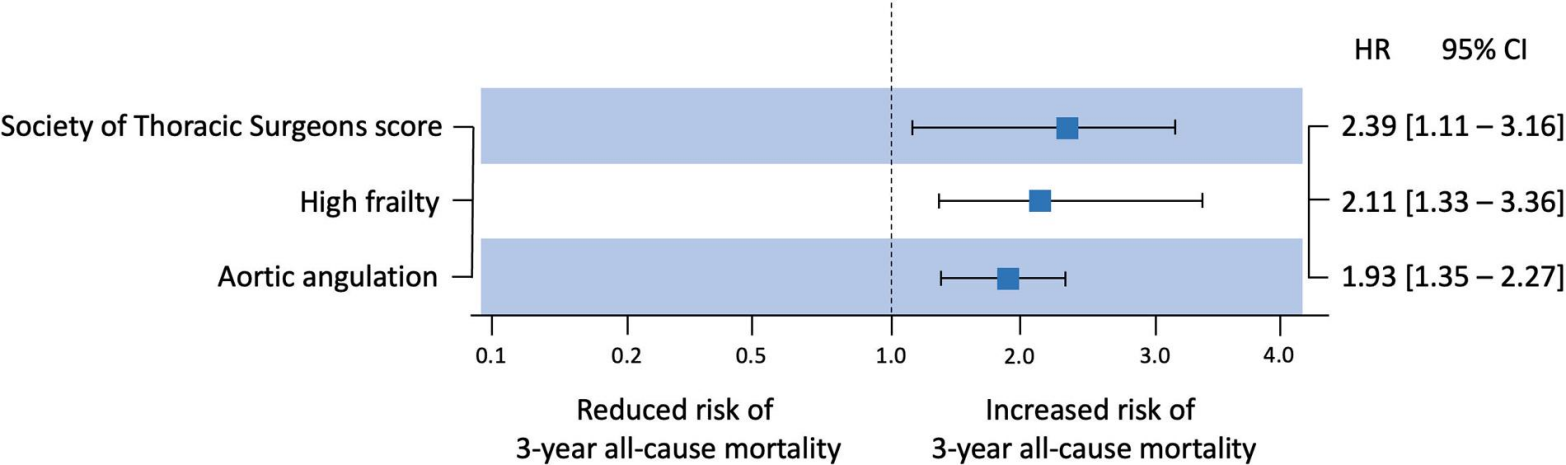
Forest plot illustrating predictive and protective factors for 3-year all-cause mortality after transcatheter aortic valve replacement.

At a median follow-up of 39.7 (IQR: 36.4–41.8) months, no difference was observed in the 3-year all-cause mortality (*P* = 0.740) or in the composite end point (*P* = 0.170) between the two populations (Figure 2). The univariate and multivariate Cox regression results for primary end points are shown in Figure 3 and Supplementary Table S4. After multivariate adjustment, three factors were independently associated with 3-year all-cause mortality: Society of Thoracic Surgeons (STS) score [hazard ratio [HR] = 2.39, 95% confidence interval [CI] = 1.11–3.16, *P* < 0.001; per 1% increase in score], high frailty (HR = 2.11, 95% CI = 1.33–3.36, *P* < 0.001; defined as frailty score above the median cutoff), and aortic angulation (HR = 1.93, 95% CI = 1.35–2.27, *P* = 0.031; per 1-degree increase). Notably, the clinical outcomes of patients with/without AS in rheumatic AR and non-rheumatic AR are shown in Supplementary Figure S1 and Supplementary Table S5. A Kaplan–Meier analysis showed no significant difference in the 3-year all-cause mortality (*P* = 0.280) or in the composite end point (*P* = 0.320) among the four subgroups.

## 4 Discussion

The present study focused on a strictly defined high surgical risk cohort with severe AR (EuroSCORE II >12% or STS score >8%), who were often ineligible for surgical aortic valve replacement (SAVR) due to advanced age (mean 71.2 years) and comorbidities (e.g., hypertension in 64.0%–68.8%, coronary artery disease in 26.4%–27.6%). Against this clinical background, we present the primary outcomes of the present cohort study, which are summarized as follows: (1) No statistically significant difference was observed in the 3-year all-cause mortality or composite endpoint between patients with rheumatic AR and non-rheumatic AR. (2) The Society of Thoracic Surgeons score, higher frailty, and larger aortic angulation were associated with 3-year all-cause mortality. (3) No statistically significant differences in the studied endpoints were found between patients with rheumatic AR and non-rheumatic AR, regardless of concurrent aortic stenosis.

Rheumatic heart disease, a cardiac sequela of acute rheumatic fever caused by type A streptococcal infection which remains a major component of valvular heart disease in low- and middle-income countries (1, 2, 4). It is estimated that 30% of patients with RHD will develop rheumatic AV disease (4), among which pure AR accounts for 14%, nearly twice that of AS (5).

At present, SAVR is still the preferred treatment for AV diseases of various etiologies (6). However, patients in developing countries have a higher burden of poorly controlled or undiagnosed chronic disease and may therefore be at relatively higher surgical risk for SAVR (15). The feasibility and safety of TAVR in RHD will provide potential therapeutic possibilities for patients with rheumatic AR in low- and middle-income countries (9). Our study is the largest feasibility study to date of TAVR in patients with rheumatic AR. Larger aortic angulation was a common risk factor for 3-year all-cause mortality and the composite end point in the current study. Considering that all patients underwent with the transapical approach, severe fibrosis and lack of calcification may amplify the challenge carried by aortic angulation, and inadequate implant depth and coaxiality accuracy may lead to poorer THV performance. Nevertheless, the absence of statistically significant differences in the key studied endpoints between the two populations is encouraging. Notably, key intraoperative complications (annulus rupture, coronary obstruction, conversion to open heart surgery) were rare and comparable between groups (Table 3), and the incidence of ≥ mild paravalvular leakage was significantly lower in the rheumatic AR group (5.6% vs. 11.3%, *P* < 0.001). Furthermore, the rates of VARC-3-defined key safety endpoints (stroke, major bleeding, vascular complications, acute kidney injury, and permanent pacemaker implantation) were similarly low and comparable between the two groups (Table 3), with no statistically significant differences observed (all *P* > 0.05). These findings collectively provide direct and comprehensive evidence of the procedural safety of TAVR in rheumatic AR patients. Recent studies have shown that mixed AV disease accounts for 7% of RHD patients (5), and TAVR appears to be reliable in such patients (16). In this study, patients with rheumatic AR and those who did not have rheumatic AR were separated into subgroups combined with or without AS, and the clinical outcomes were compared and evaluated. The results showed no significant difference in 3-year all-cause mortality or composite end point among the four subgroups, which is encouraging. Notably, previous stroke and high frailty were predictors of the primary end point, which may provide guidance for the cardiac team to comprehensively assess the patient's functional status before undergoing the procedure and to formulate a surgical plan.

This study has some limitations. First, due to the limited sample size, we did not use propensity score matching overlapped with a weighted analysis to adjust for measured confounding factors. Second, the transapical approach used in this study was dominant, which may lead to insufficient extrapolation of the study results. Third, the study included relatively young patients, and fewer comorbidities with a lower risk of having the procedure could have implications for the clinical applicability of the findings. Finally, this observational non-randomized study has unmeasured confounding, findings only show associations, and outcome similarity conclusions are limited to the studied endpoints.

## 5 Conclusion

Patients with rheumatic AR exhibited procedural and clinical outcomes similar to those of patients with non-rheumatic AR. These findings suggest that TAVR may offer a feasible solution for addressing the complex pathophysiological characteristics of the aortic root in such patients, while also providing foundational support for future studies.

## Data Availability

The datasets presented in this study can be found in online repositories. The names of the repository/repositories and accession number(s) can be found in the article/Supplementary Material.
