# Supplementary material for: Safety and efficacy of transcatheter aortic valve replacement in rheumatic aortic regurgitation: a prospective cohort study
Source: Front Cardiovasc Med. 2026 Feb 18;13:1662827. doi: 10.3389/fcvm.2026.1662827 (PMC12957259; doi:10.3389/fcvm.2026.1662827)
Supplement: Supplementary file 1 [file Datasheet1.docx]

**SUPPLEMENTAL DATA**

**
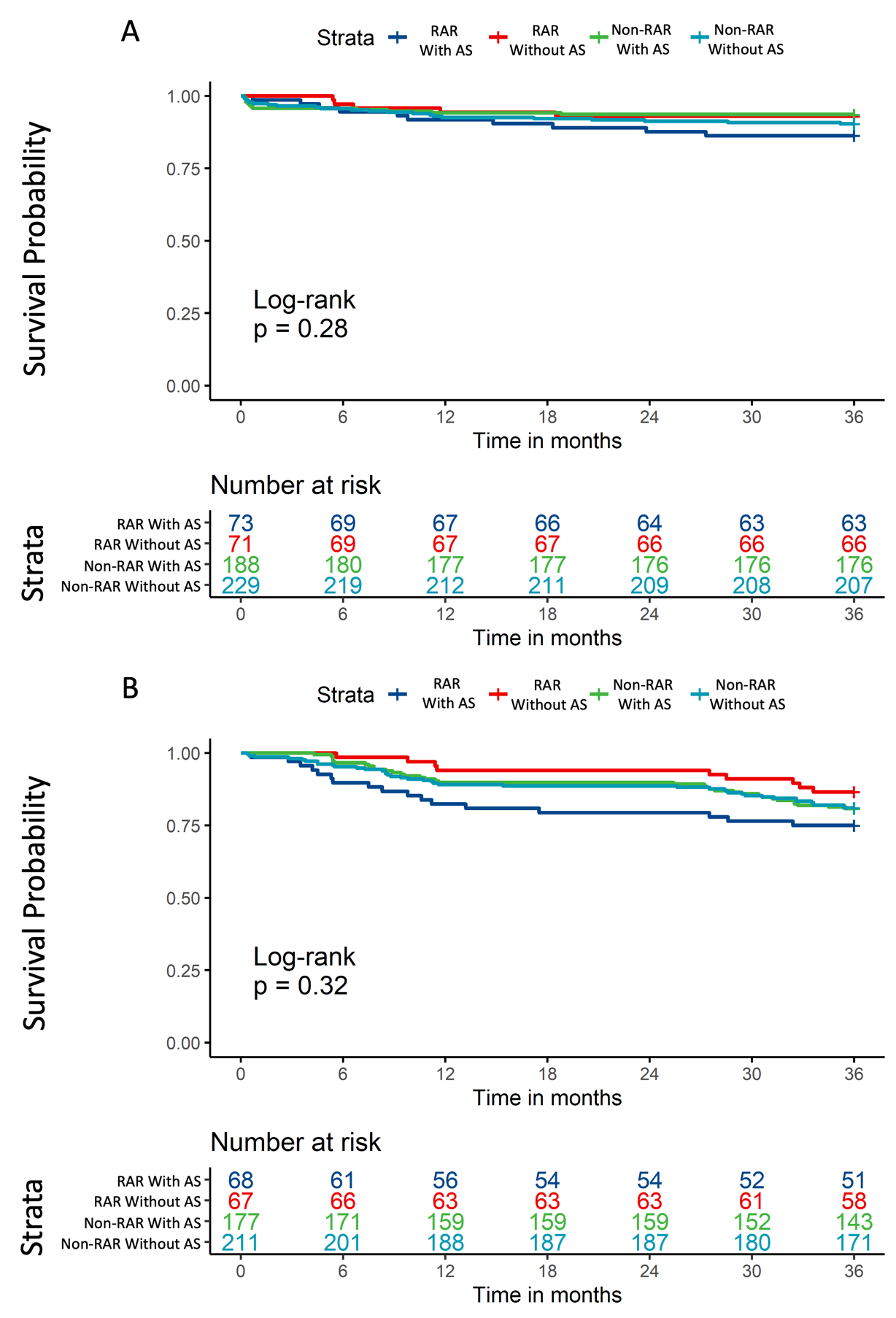
**

**Figure S1** Kaplan-Meier analysis of all-cause mortality (A) and composite end point (B) at 3 years comparing patients with rheumatic AR with AS, rheumatic AR without AS, non-rheumatic AR with AS, and non-rheumatic AR without AS. AS, aortic stenosis; RAR, rheumatic aortic regurgitation.

**Table** **S1** Baseline Characteristics of Patients of Aortic Regurgitation Combined with Aortic Stenosis

|  | Non-rheumatic AR-TAVR (n = 188) | Rheumatic AR-TAVR (n = 73) | *P* Value |
| --- | --- | --- | --- |
| Age, yrs | 71.0 (66.0 - 74.5) | 74.0 (66.0 - 80.0) | 0.062 |
| Male | 73.9 (139) | 38.4 (28) | **< 0.001** |
| BMI, kg/m^2^ | 23.2 (21.1 - 24.6) | 25.2 (22.5 - 28.2) | **< 0.001** |
| BSA, m^2^ | 1.7 (1.6 - 1.7) | 1.8 (1.6 - 1.9) | **< 0.001** |
| NYHA functional class III or IV | 98.4 (185) | 95.9 (70) | 0.291 |
| STS score, % | 7.4 (5.4 - 9.6) | 8.5 (6.3 - 9.9) | 0.064 |
| EuroSCORE, % | 11.2 (7.0 - 14.0) | 9.1 (6.9 - 10.7) | 0.074 |
| Diabetes mellitus | 18.6 (35) | 28.8 (21) | **< 0.001** |
| Hypertension | 62.8 (118) | 91.8 (67) | **< 0.001** |
| Dyslipidemia | 17.6 (33) | 27.4 (20) | **< 0.001** |
| Cerebrovascular disease | 2.1 (4) | 1.4 (1) | N/A |
| Coronary artery disease | 27.1 (51) | 24.7 (18) | 0.369 |
| PCI | 3.2 (6) | 12.3 (9) | **< 0.001** |
| CABG | 0.5 (1) | 8.2 (6) | **0.002** |
| Atrial ﬁbrillation | 19.7 (37) | 32.9 (24) | **< 0.001** |
| Previous PM | 2.1 (4) | 0 (0) | N/A |
| Peripheral artery disease | 38.8 (73) | 34.2 (25) | 0.586 |
| NT-proBNP | 2389.5 (1746.5 - 3017.5) | 1969.0 (1447.0 - 3070.0) | 0.153 |
| Frailty score | 10.5 (8.3 - 12.9) | 8.2 (5.9 - 16.7) | 0.068 |
| High frailty | 12.2 (23) | 27.4 (20) | **0.005** |

Values are expressed in interquartile range, or % (n). The p values in **bold** represent differences between groups with p values < 0.05.

BMI, body mass index; BSA, body surface area; PCI, percutaneous coronary intervention; CABG, coronary artery bypass graft; PM, pacemaker; N/A, not applicable; NYHA, New York Heart Association; STS, Society of Thoracic Surgeons; NT-proBNP, N-terminal pro–brain natriuretic peptide.

**Table** **S2** Baseline Echocardiographic and Computed Tomography Angiography Characteristics of Patients of Aortic Regurgitation Combined with Aortic Stenosis

|  | Non-rheumatic AR-TAVR (n = 188) | Rheumatic AR-TAVR (n = 73) | *P* Value |
| --- | --- | --- | --- |
| **Transthoracic echocardiography** | | | |
| BAV | 6.4 (12) | 24.7 (18) | **< 0.001** |
| Mean pressure gradient, mmHg | 9.9 (8.1 - 11.9) | 14.2 (12.8 - 16.3) | **< 0.001** |
| LVEF, % | 51.0 (44.0 - 56.0) | 47.0 (44.0 - 57.0) | 0.638 |
| ≥ moderate MR | 22.9 (43) | 23.3 (17) | 1.000 |
| **Computed tomography angiography** | | | |
| Aortic annulus area, mm^2^ | 587.1 (518.8 - 638.1) | 548.0 (504.0 - 599.0) | 0.124 |
| Aortic annulus diameter, mm | 27.8 (26.4 - 28.9) | 27.0 (26.2 - 28.5) | 0.405 |
| LVOT diameter, mm | 29.4 (27.4 - 30.5) | 27.9 (24.8 - 30.4) | **0.007** |
| STJ diameter, mm | 34.4 (31.4 - 36.7) | 32.6 (30.6 - 35.9) | **0.032** |
| AA diameter, mm | 38.7 (36.0 - 40.6) | 37.0 (34.9 - 40.3) | **0.021** |
| EOA, cm^2^ | 1.0 (0.8 - 1.2) | 1.0 (0.8 - 1.1) | 0.087 |
| EOAi, cm^2^/ m^2^ | 0.9 (0.7 - 1.0) | 0.9 (0.7 - 1.0) | 0.179 |
| Aortic angulation, degree | 43.0 (39.0 - 50.0) | 45.0 (41.0 - 50.0) | 0.282 |

Values are expressed in interquartile range, mean ± SD, or % (n). The p values in **bold** represent differences between groups with p values < 0.05.

AA, ascending aorta; AS, aortic stenosis; BAV, bicuspid aortic valve; EOA, effective orifice area; EOAi, indexed EOA; FAC, fractional area change; LVEF, left ventricle ejection fraction; LVOT, left ventricular outflow tract; MR, mitral regurgitation; PPM, prosthesis-patient mismatch; STJ, sinotubular junction.

**Table** **S3** Peri-procedural Characteristics of Patients of Aortic Regurgitation Combined with Aortic Stenosis

|  | Non-rheumatic AR-TAVR (n = 188) | Rheumatic AR-TAVR (n = 73) | *P* Value |
| --- | --- | --- | --- |
| **Bioprosthetic valve size** | | | |
| < 25 mm | 11.7 (22) | 26.0 (19) | **< 0.001** |
| ≥ 26 mm | 88.3 (166) | 74.0 (54) | **< 0.001** |
| Implanted valve oversize | 8.5 (16) | 17.8 (13) | **< 0.001** |
| **Intraprocedural characteristics** | | | |
| Conversion to open heart surgery | 2.1 (4) | 1.4 (1) | N/A |
| Annulus rupture | 2.1 (4) | 1.4 (1) | N/A |
| Coronary obstruction | 0.5 (1) | 0 (0) | N/A |
| Valve-in-valve implant | 0.5 (1) | 2.7 (2) | 0.393 |
| ≥ Mild paravalvular leakage | 14.9 (28) | 8.2 (6) | **< 0.001** |
| Operation time, min | 120.0 (105.0 - 127.5) | 117.0 (94.0 - 139.0) | 0.947 |
| Fluoroscopy time, min | 8.6 (6.3 - 10.5) | 7.9 (5.9 - 10.5) | 0.692 |
| **Periprocedural outcomes** | | | |
| Stroke | 2.1 (4) | 2.7 (2) | 1.000 |
| Life-threatening bleeding | 2.1 (4) | 0 (0) | 0.581 |
| Major vascular complications | 5.3 (10) | 4.1 (3) | 1.000 |
| Acute kidney injury Staging ≥ 3 | 3.7 (7) | 6.8 (5) | 0.315 |
| New-onset permanent pacemaker implantation | 6.4 (12) | 5.5 (4) | 1.000 |

Values are expressed in interquartile range, or % (n). The p values in **bold** represent differences between groups with p values < 0.05.

N/A, not applicable; TAVR, transcatheter aortic valve replacement; DSA, digital subtracted angiography.

**Table** **S4** Univariate and Multivariate Cox Regression Analyses of Factors Associated with 3-Year All-Cause Mortality in Overall Population

| **Characteristics** | **Univariate Analysis** | | | **Multivariate Analysis** | | |
| --- | --- | --- | --- | --- | --- | --- |
|  | **Hazard ratio** | **95% CI** | ***P-*value** | **Hazard ratio** | **95% CI** | ***P-*value** |
| BMI | 1.21 | 1.09–1.33 | < 0.001 |  |  |  |
| STS score | 2.53 | 1.43–4.46 | 0.001 | 2.39 | 1.11–3.16 | < 0.001 |
| Diabetes mellitus | 1.31 | 1.19–1.43 | < 0.001 |  |  |  |
| Cerebrovascular disease | 2.33 | 1.15-3.69 | < 0.001 |  |  |  |
| High frailty | 5.00 | 2.86-8.77 | < 0.001 | 2.11 | 1.33–3.36 | < 0.001 |
| Aortic annulus diameter | 1.09 | 1.06-1.22 | < 0.001 |  |  |  |
| Aortic angulation, degree | 1.98 | 1.66-2.57 | < 0.001 | 1.93 | 1.35–2.27 | 0.031 |

BMI, body mass index; STS, Society of Thoracic Surgeons; PPM, prosthesis-patient mismatch.

**Table** **S5** Univariate and Multivariate Cox Regression Analyses of Factors Associated with 3-Year All-Cause Mortality in Patients of Aortic Regurgitation Combined with Aortic Stenosis

| **Characteristics** | **Univariate Analysis** | | | **Multivariate Analysis** | | |
| --- | --- | --- | --- | --- | --- | --- |
|  | **Hazard ratio** | **95% CI** | ***P-*value** | **Hazard ratio** | **95% CI** | ***P-*value** |
| BMI | 1.26 | 1.08–1.46 | 0.002 |  |  |  |
| STS score | 2.69 | 1.51-3.99 | 0.004 |  |  |  |
| Diabetes mellitus | 3.35 | 1.38-6.13 | 0.007 |  |  |  |
| Cerebrovascular disease | 2.74 | 1.07-3.95 | 0.002 | 1.53 | 1.30-1.86 | 0.009 |
| Peripheral artery disease | 1.63 | 1.36-2.62 | < 0.001 |  |  |  |
| High frailty | 5.20 | 2.08-9.13 | < 0.001 | 3.05 | 1.12–7.22 | < 0.001 |
| Aortic annulus diameter | 1.19 | 1.04-1.21 | 0.001 |  |  |  |
| Aortic angulation, degree | 1.09 | 1.05-1.14 | < 0.001 |  |  |  |

AS, aortic stenosis; BMI, body mass index; STS, Society of Thoracic Surgeons.
